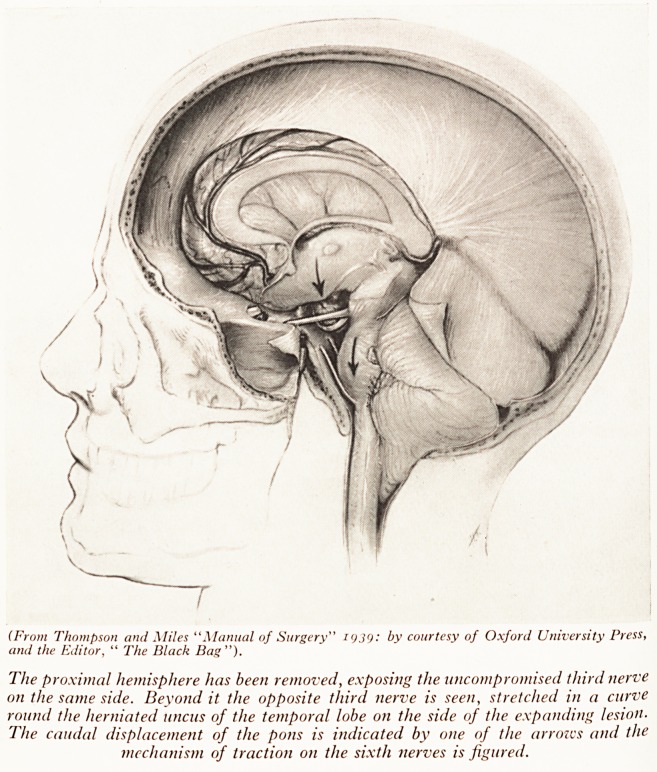# Head Injuries
*A paper given to the Bristol Medico-Chirurgical Society, 13th March, 1957.


**Published:** 1957-07

**Authors:** G. L. Alexander

**Affiliations:** Neurosurgeon, Frenchay Hospital, Bristol


### HEAD INJURIES*

BY

G. L. ALEXANDER, F.R.C.S.

*Neurosurgeon, Frenchay Hospital, Bristol*

The management of cases of head injury has been improving since the turn of the century; but in this mechanical age their incidence has risen steeply.

One major advance in our understanding of the mechanism in head injury we owe to the writings of Sir Geoffrey Jefferson before the War. He brought to general notice the importance of midbrain dysfunction in the causation of unconsciousness, not only following trauma but also in cases of tumour and abscess of the brain. This is how it comes about. When one cerebral hemisphere is displaced medially—as by clot on the convexity, or by pus or fluid, for that matter—or when it expands by reason of a swelling within it, only a limited amount of room to contain its increasing volume can be afforded by bulging across the midline under the falx. The only other available direction for expansion is downwards through the tentorial aperture. The innermost part of the temporal lobe, adjacent to the midbrain, becomes squeezed round the free margin of the tentorium, forming a hernial ridge which compresses the midbrain and also stretches the third cranial nerve. The brain, then, has to expand downwards through the tentorial aperture. When it does so it also pushes the midbrain and pons caudally. The effect of this movement is to stretch the many small arteries passing in to nourish the midbrain and pons. The basilar artery is tethered by the posterior cerebral arteries, and its caudal movement is therefore limited. The midbrain and pons suffer an ischaemia and consciousness is lost.

### MIDBRAIN HAEMORRHAGE

I have dwelt a little on the morbid anatomy of these displacements of the brain, which incidentally are common to all types of expanding supratentorial lesion, because the next step in the story is an irretrievable disaster. Plate VII shows the disaster—acute softening and haemorrhages within those vital parts of the brain. We know that it can occur quite suddenly, revealed in those cases of frontal extradural or intracerebral clot, and occasionally of abscess too, where the patient remains conscious, until he plunges into coma. Nothing can save the patient with haemorrhages in the brain stem. But in most cases there is ample time for investigation and intervention, provided the earliest clinical signs of impending danger are heeded. More of that later.

It is a common misconception that a high pressure within the head causes drowsiness or unconsciousness. If saline be injected into the lumbar theca experimentally to produce a pressure of 1400 mm. or more, the chief symptom, if any, is dizziness. That is a pressure far exceeding those found even in the desperate cases.

Other factors in the improving state of affairs in the handling of cases of head injury have been, first the additions to the operating facilities in the country as a whole, whereby it is easier for burr holes to be made in the skull when there is doubt. Secondly, the introduction of arteriography has been a great advance. Arteriograms show the displacements of arteries occasioned by the presence of clots or of post-traumatic cerebral oedema. They give precision to diagnosis, or to what has perhaps been mere clinical conjecture. This applies particularly to those patients who are unconscious and quite "inaccessible" from the moment of injury. They have cerebral lacerations or severe contusion and may be suffering from the mounting effects of intracranial bleeding or of swelling of the brain. In either event the outcome is likely to be fatal

* A paper given to the Bristol Medico-Chirurgical Society, 13th March, 1957.

unless operation is performed for evacuation of clot or to provide a generous decompression. Those cases used to languish and perish, often with the label of "cerebral irritation".

With the ophthalmoscope one can see at once if the intracranial pressure is raised, because the retinal veins can be seen to be engorged. It takes a day or two for papilloedema to develop, and very often that time is not granted. This venous engorgement of the retina is probably an immediate indicator of mounting intracranial pressure.

In hospital the intracranial pressure can be measured by lumbar puncture and manometry, but this can be a dangerous procedure if a large expanding lesion be present and the cerebellar tonsils are consequently jammed down into the foramen magnum. Indeed in those circumstances a falsely low manometer reading may be obtained. The ophthalmoscope is helpful in sorting out those cases in which lumbar puncture is certain to be safe.

But lumbar puncture may have to be used as a treatment for high intracranial pressure in some cases after a head injury, when the cerebrospinal fluid contains blood. In such cases it is presumed that red cells get wafted towards the Pacchionian villi in the pulsating slow current of subarachnoid fluid, and that they pack around the villi, interfering temporarily with the resorption of the cerebrospinal fluid into the venous blood-stream. Lumbar puncture relieves the severe headaches of which these patients complain, but it is wise to know beforehand from an arteriogram that no clot or swelling of the brain is present.

### THE NATURE OF CONCUSSION

Some remarks are appropriate now on the subject of simple concussion, that brief reversible coma, unassociated with structural damage to the brain. Concussion remains a mystery. It would be true to say that we have no more concise ideas as to its nature than our medical ancestors who in their Latin texts called it *commotio cerebri*.

Professor Dott of Edinburgh has recently advanced a suggestion which may well be right on the target; I am greatly indebted to him for allowing me to mention his hypothesis.

First of all I should say that cerebral arteries resent stretching, and respond by contracting vigorously. Some clinicians still find it hard to believe that the calibre of the intracranial arteries is variable; the neurosurgeon sees this occasionally to a quite dramatic degree.

The second point supporting the argument concerning the theory of concussion is that the unconsciousness of subarachnoid haemorrhage due to rupture of an aneurysm at or near the circle of Willis is almost certainly due to spasm of the perforating arteries which run up from the circle *via* the perforated spots into the base of the brain. We deduce this from studies of the territories of red softening in the diencephalon, apparent in fatal cases of subarachnoid haemorrhage, devoid of thrombosis in the arteries supplying the softened territory; this is an important observation. The diencephalon is another "seat of consciousness", to put it rather colloquially.

A further point is that the timing of the unconsciousness in very many cases of spontaneous subarachnoid haemorrhage is just right for the onset of a vascular spasm. How often do we hear that the patient had time to cry out "Oh my head" before he or she drops to the ground. Moreover, the volume of blood which escapes from the aneurysm is in so many cases patently far too small to cause unconsciousness and death.

Professor Dott postulates that a shearing strain is imposed on those small perforating arteries at the base of the brain by a concussional blow on the head, and that the vessels promptly go into spasm. The quite surprising mobility of the brain within the cranium certainly permits such a hypothesis. His postulate of a temporary diencephalic ischaemia could account for the clinical features of simple concussion and for its variably rapid reversibility.

### RADIOLOGICAL EXAMINATION

In these days it is perhaps unnecessary to stress the importance of radiological examination of the skull. The benign-looking scalp wound in a patient who has perhaps not even been stunned, may have a depressed fracture underlying. Sometimes little tufts of hair get trapped in a fissure fracture, which must have received the hair when the crack was sprung open at the moment of impact. Cases like this, treated casually, can lead to endless trouble and danger with osteitis and its intracranial sequelae.

Many years ago a patient was admitted to a ward of my previous hospital following a car accident. She had been dazed for only a very short time, and had only a few abrasions. I had to insist that the skull be examined radiologically before the patient be allowed to depart. The film showed an upholsterer's tack driven squarely into one parietal bone, and with not a drop of blood to show for it on the scalp.

Medico-legal attention is far too much focussed on the supposed importance of fracture of the skull. Undoubtedly this stems from the obvious importance of fractures in the limbs and trunk, in regard to weight-bearing and other uses of the limbs. Of course one cannot belittle the fracture which could provide a pathway for intracranial spread of infection.

Here is another story. Some years ago a man sustained a head injury and had an unimportant but clinically-obvious fracture of the base of the skull anteriorly. He was a known bleeder but his blood seemed normal with all tests then current. He developed an intracerebral haematoma. We operated, and as we had warned the relatives might well be expected, the haematoma recurred in spite of the most careful haemostasis. Eventually we lost this patient after re-operation and transfusions, and the relatives were satisfied that we had done everything possible. At the inquest the main cause of death was given as fracture of the base of the skull. The family were aghast, and obtained free legal aid to try to make out a case of negligence. The X-rays, including our arteriograms, have I suspect been around the country to various experts —all at the public expense—for someone perhaps to detect the invisible fracture which was of such negligible clinical importance in the case.

*Per contra*, one sees cases with a mosaic of fissures criss-crossing over the vault. I can say from long experience that among those surviving for admission to a neurosurgical service, it is rare to have to operate for intracranial mischief in cases of this type, and one after the other they recover—most of them completely. One must presume that much of the force of impact is expended in fragmenting the skull, and that some favourable deceleration of the brain can give it some protection.

So much for the financial value of fractures of the skull. It may be objected that the presence of a fracture is an index of the severity of injury, but it is an inconstant and unreliable index. Some of the worst post-traumatic disabilities follow injuries without fracture. The attention of legal assessors ought to be concentrated on the story of the injured person's unconsciousness and its sequelae, and on the retrograde and post-traumatic amnesia.

### CHRONIC SUBDURAL HAEMATOMA

I think it appropriate to say a little about chronic subdural haematoma. I wish first to explode the idea that a history of relevant head injury is usually forthcoming in these cases. In fully half of the cases I have handled, no knock on the head was known to have occurred. Many of these patients, seemingly with a reasonably good recollection of events prior to operation, remember the pertinent injury as they are about to go home from hospital, or tell us about it at a follow-up appointment. Often the causative blow on the head is seemingly trivial, and is therefore forgotten.

The next point worth emphasis, is the frequency with which *fluctuation in the level of consciousness* is observed in this condition. This is an important diagnostic feature; and it has a plausible explanation. The most gross examples of tentorial herniation are to be seen in the presence of a chronic subdural haematoma, because time is afforded for a gradual deformation of the parts. I have seen part of the temporal horn of the ventricle in one of these herniations. Now, compression of the midbrain must impair the flow of fluid down the aqueduct, and the lateral and third ventricles dilate. It is probable that fluid is forced past the obstruction intermittently, which would accord with the frequently-varying grades of consciousness so characteristic of chronic subdural haematoma.

In earlier years, before the War, we used to have an unexplainably high mortality with these cases. We would let out a large volume of dark brown fluid, the unconscious patient would recover in the theatre—and perhaps even ask for a cigarette (which note you, in those carefree days, was provided *instanter*—match and all!) Next day drowsiness would recur, and by midnight perhaps we would be at him again with brace and burr. Some more fluid would be released, with a shorter and less effective reprieve; but surely he died. It was all a bit odd, and highly frustrating. Mr. Dott had us revive the Boston practice in Dr. Cushing's clinic, of perfusing the carotid arteries with formol-saline some hours before the autopsy. This procedure of partial fixation of the brain *in situ* reveals the distortions and displacements of the brain to be far more gross than is apparent when the brain is removed in the unhardened state.

Returning to our problem, there was the solution plain to see—the unrelieved temporal lobe herniation and acqueduct obstruction, with secondary dilatation of the ventricles.

Our mortality rate dropped abruptly when we drained one lateral ventricle continuously into a glass flask by catheter and Southey's tubing for a few days to give time for disimpaction of the herniation to take place. A much more direct and simple remedy was proposed by Lalonde and Gardner of Cleveland (1948)—merely to push the herniated ridge of temporal lobe from below upwards by injecting saline solution into the lumbar theca. This is dramatically effective.

In this connection, there is an old and true adage concerning cases of extradural haemorrhage, that if the brain does not "expand" when the clot has been evacuated, the patient will die. In reality there is nothing of expansion in the situation; the brain is held in its displaced position because the temporal lobe is nipped in the tentorial aperture and cannot come up to its normal level in contact with the skull. This trick of intrathecal saline injection has diminished the mortality in cases of extradural haematoma also.

### THE SIGNS OF DANGER IN HEAD INJURY

I come now to the "meat" of my discourse. It concerns the suggestions I should like to offer for guidance in suspecting that trouble may be on the way in a case of head injury.

I think the commonest, earliest and most significant sign is a *deterioration in the state of consciousness*. The lucid interval syndrome of extradural haemorrhage is a classical example: first the concussional unconsciousness, then recovery—and often complete recovery—then headache and probably vomiting, drowsiness and then unconsciousness, as the midbrain distortion develops.

But what about the patient with a contused brain and perhaps with a laceration, who is unconscious from the start? He too shows a lessening of responsiveness, if he is running into trouble. Incidentally, I recommend firm rubbing of the skin over the sternum as a most effective means of penetrating the screen of unconsciousness.

Another important clue is the appearance *de novo* of neurological abnormalities, or the increase of existing deficits, such as paresis, changes in reflexes, or dysphasia if the patient has previously been able to speak. This may signify that an intracerebral clot is enlarging or that oedema is developing around an injured area of the brain.

*Dilatation of the pupil* on the side of the expanding lesion is an ominous sign, universally familiar. Plate VIII shows why this occurs. Once again the herniation of the temporal lobe is the cause of the trouble. If both pupils dilate and become inactive to

PLATE VII

*A section through the midbrain showing widespread punctate and diffuse haemorrhages, which were the consequence of unrelieved supratentorial hypertension and caudal displacement of the brain stem.*

PLATE VIII

*(From Thompson and Miles "Manual of Surgery" 1939: by courtesy of Oxford University Press, and the Editor, " The Black Bag ").*

*The proximal hemisphere has been removed, exposing the uncompromised third nerve on the same side. Beyond it the opposite third nerve is seen, stretched in a curve round the herniated uncus of the temporal lobe on the side of the expanding lesion. The caudal displacement of the pons is indicated by one of the arrows and the mechanism of traction on the sixth nerves is figured.*

light soon after injury, the midbrain may be assumed to have gone out of commission, and the patient's fate is sealed.

*Restlessness* is a common accompaniment of intracranial hypertension. There is a quality about this restlessness which becomes familiar to us who see many of these patients, but which I find rather hard to describe. It is intermittent though frequent, and has a sort of frantic determination about it. A full bladder is, of course, another and banale cause of restlessness; and retention of urine is not uncommon after injury to the head.

Another portent of danger is a *deepening of respiration.* I do not refer to the familiar deep, rapid and noisy breathing which so often advertises to the inmates of a ward that serious events are afoot behind the screens. You must all have seen the patient who has a pink suffusion of his face and who is breathing as if he had been engaged in some moderate exertion. He looks hot—and in the vernacular, he certainly is a "hot" case.

It would, I think, be redundant for me to do more than enumerate the other classical accompaniments of severe intracranial hypertension; the rise of blood pressure, to keep the cerebral circulation going in a compressed brain; the slowing of the pulse and its ante-mortem acceleration; the slowing of respiration and its ante-mortem acceleration. Cheyne-Stokes respiration is of ill omen; when occurring early, it indicates that a mortal injury was sustained at the time of the accident. If it appears in a case for which it seems that something might still be done, it usually means that matters have gone too far for any effective treatment.

### THE SELECTION OF CASES FOR NEUROSURGICAL TREATMENT

I should like now to raise the question of the management of head injuries in a topographical sense.

In the first place only about one in eight cases of acute head injury admitted to hospital requires opening of the head, either by burr holes or by craniotomy.

Circumstances beyond our control have enforced a "vetting" of cases for transfer to the neurosurgical service in Bristol. We have had to ask to be allowed the opportunity of discussion by telephone before accepting cases. We welcome this opportunity of expressing our gratitude to the general practitioners especially and also the consultants, in the Region, for the apparent willingness with which they have put up with this innovation. Of the 650 or more cases admitted annually to the Unit, about 400 are emergencies—or "urgencies"—admitted within one or two days: and of these acute cases only one in five is a case of head injury. We are greatly helped also by the readiness of consultants to accept back their cases for completion of convalescence or if the case is inoperable; but all concerned would be more happy were we able to "see our own jobs through".

Extradural haematoma is not at all rare—we have twelve to sixteen of them a year. Acute subdural haematoma occurs more frequently, and swelling of the traumatised brain requiring decompression, less often. Some years ago a questionnaire was sent round by me to other neurosurgical centres, using the incidence of cases of extradural haemorrhage as a yardstick. The incidence in Bristol was three to ten times higher than in nearly all of the other centres. Now I cannot believe that this Region is a particularly dangerous one in which to live, and I am led to the conclusion that those telephone consultations must be bringing along the cases.

Mr. Phillips and Mr. Hulme have been looking into our archives and several pertinent facts emerge. The first is the conspicuously low mortality-rate among the cases of extradural haemorrhage: in some recent years it has been as low as 10 per cent. In America it is about 45 per cent. in neurosurgical centres, and around 75 per cent. in general hospitals (Kahn *et al.*, 1955). These figures do not reflect on the standards of diagnosis and treatment, but bear directly on what goes on before the patient reaches hospital, and how quickly action is taken. The alert practitioner—and we seem to have a lot of them around—the houseman who is "on his toes" and a generally commendable ambulance service, are all playing the major part in the results.

Sometimes, on discussion, it has been very evident that operation must proceed forthwith at the peripheral hospital.

The second point which Mr. Hulme has brought out in his enquiries relates to the end-results in acute subdural haematoma. Because of the associated contusion and laceration of the brain, the proportion of complete recoveries, in the very few published series, has been notoriously low. The return-to-work rate in our cases has been improving over the years, and is now around 35 per cent.—a very good comparative figure. There can be no doubt that this beneficent trend follows from our receiving these cases earlier, and I think the telephone is helping in this. The results in cases of extradural haemorrhage present a contrast; nearly all recover fully. They are therefore economically the more rewarding.

Some apology is required for the note of domestic advertising in those comments on our experience in Bristol, but the evidence presented indicates directions in which improvements could be effected. There is no magic in what we do for those head-injured. It is traditional treatment and some of it was carried out successfully in general hospital services—and still is—throughout the country.

### NEUROSURGERY AND THE GENERAL SURGEON

Having regard to the fact that most of our cases in which a major intracranial lesion has been revealed at operation have come from this area, within a radius of thirty miles or so of Bristol, the question arises, what of the similar cases at a distance from a neurosurgical centre? My own view is that ideally in each major centre of surgery at a distance from a neurosurgical unit it would be desirable for two of the general surgeons to be versed in the tricks of the trade, particularly in the staying of haemorrhage during craniotomy, and in haemostasis of the brain.

It is no longer sufficient to rely on the classical subtemporal craniectomy for middle meningeal haemorrhage. One third of the clots we evacuate are frontal or parietal, and would be missed by the classical operation, which is also often useless and dangerous in the other conditions requiring decompression.

Even more to the point for the surgeon is the acquisition of speed in the preparation of the scalp and in raising a bone flap, for the patient's sake at times, and for the sake of the theatre and other staff at all times. It is largely a matter of providing good razors, proper bone-cutting tools, diathermy of a quality suitable for intracranial haemostasis, and a sucker which will stand up to hard work. But there is more to it than operating. The diagnostic side would need development, and in that arteriography would play a part. Cerebral angiography is spreading. It has ceased to be the monopoly of the neurosurgeon, now that its technique has been radically simplified.

I can see all sorts of difficulties in trying to implement a policy of development of some neurosurgical knowledge and skill at the periphery, even were the policy accepted in principle. The optimum grade in which a training of several months could be given would I think be in that of senior registrar. But then the difficulty arises of ensuring a distribution of those trained surgeons. Perhaps secondment to a neurosurgical unit, for the requisite time, on appointment (in advance) as a general surgeon would overcome the difficulty. I must emphasize that a casual or brief introduction to neurosurgical diagnostic and operative techniques would be ineffective in its purpose and potentially dangerous. Further, the suggested policy should apply only to those cases of such urgency that time will not permit of their transfer to a special unit.

I see no difficulty in disseminating knowledge about better after-care, which is dominantly a matter of preventing inhalation and infection in the airways.

### PREVENTION

I should like to draw attention to a book which records the proceedings of the first Congress on the Medical Aspects of Traffic Accidents, held in Montreal in 1955. The contributors are from Canada, the United States and this country. It is published under the aegis of a Trust Foundation in Canada. Copies have been given to medical libraries (including that of Bristol University). Slow-motion film analyses of what happens to car and occupants in different types of crashes are described. The anatomy of wrecked cars is studied, as are tests of safety devices and of radical alterations in car design and in fittings. The law relating to "drunk-in-charge", and tests for drunkenness, are discussed in the light of the various practices in different states and countries. And as an example of other topics, the difficult question of the controlled epileptic and his driving again.

I noticed recently in Montreal that the driver and passengers fastened the aeroplane type of safety-belt which was fitted to the seats in many of the private cars, even for short journeys in the city.

The high cost to the individual of hospital treatment in the New World, which commences with a charge for the ambulance (complete with intern and orderlies) from the scene of the accident, must be an incentive to lessen the toll of the roads. A costly draft on an annual benefit under personal sickness insurance, may leave a debt with nothing to cover illness. Damages awarded in the courts may not be recovered for years, if at all. In contrast, we are fortunate in this country, with our hospital service which has *always* been free; and with our compulsory third-party insurance which has no ceiling on company's liability, and is not therefore tied to the annual premium the driver elects to pay, as in the New World.

It is of interest that we have observed a substantial drop in the incidence of cases from motor-cycle accidents admitted to Frenchay Hospital since the wearing of crash-helmets became common. As might be expected, our plastic-surgeon colleagues have not noticed any lessening of their load of facial injuries; indeed some of the injured are probably now being spared from death at the roadside, and reach hospital.

A large dividend can be expected from research into the causes of accidents and into ways and means of minimizing the injuries received in them. If due regard is paid to the latter factor we shall have to accept some sacrifice of style and elegance in the motor-car of the future. The brain of civilized man is becoming more widely of importance to his earning capacity and to his place in society. It behoves us to place the conservation of brains in the forefront of our considerations.

### REFERENCES

Jefferson, G. (1938). *Arch. Neurol. and Psych.*, **40**, 857.

LaLonde, A. A., and Gardner, W. J. (1948). *New Eng. J. Med.*, **239**, 493.

Kahn, E. A., Bassett, R. C., Schneider, R. C. and Crosby, E. C. *Correlative Neurosurgery*. C. C. Thomas, Springfield, Ill., U.S.A. 1955.

## Figures and Tables

**Figure f1:**
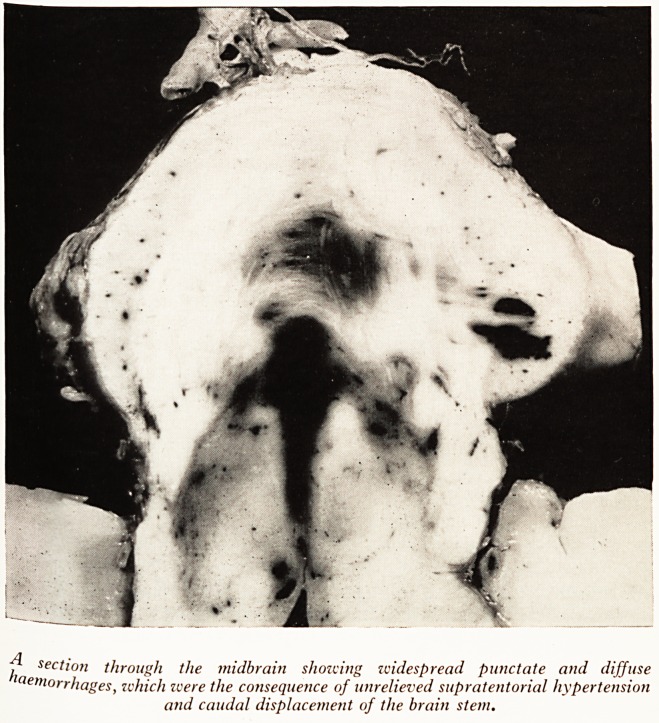


**Figure f2:**